# From Genetics to Epigenetics, Roles of Epigenetics in Inflammatory Bowel Disease

**DOI:** 10.3389/fgene.2019.01017

**Published:** 2019-10-31

**Authors:** Zhen Zeng, Arjudeb Mukherjee, Hu Zhang

**Affiliations:** ^1^Department of Gastroenterology, West China Hospital, Sichuan University, Chengdu, China; ^2^Center for Inflammatory Bowel Disease, West China Hospital, Sichuan University, Chengdu, China; ^3^West China School of Medicine, Sichuan University, Chengdu, China

**Keywords:** epigenetic modifications, inflammatory bowel disease, disease susceptibility, disease activity, disease behaviour, colorectal cancer, therapeutic translation

## Abstract

Inflammatory bowel disease (IBD) is a destructive, recurrent, and heterogeneous disease. Its detailed pathogenesis is still unclear, although available evidence supports that IBD is caused by a complex interplay between genetic predispositions, environmental factors, and aberrant immune responses. Recent breakthroughs with regard to its genetics have offered valuable insights into the sophisticated genetic basis, but the identified genetic factors only explain a small part of overall disease variance. It is becoming increasingly apparent that epigenetic factors can mediate the interaction between genetics and environment, and play a fundamental role in the pathogenesis of IBD. This review outlines recent genetic and epigenetic discoveries in IBD, with a focus on the roles of epigenetics in disease susceptibility, activity, behavior and colorectal cancer (CRC), and their potential translational applications.

## Introduction

It is widely acknowledged that IBD is an extremely complicated disease with an unclear pathogenesis. Crohn’s disease (CD) and ulcerative colitis (UC) are the most common subtypes of IBD. It predominantly affects the gastrointestinal tract (GI), and results in repeated abdominal pain, diarrhea, bloody purulent stool, and weight loss, which substantially reduces the quality of life and increases the economic burden of IBD patients (Kaser et al., 2010). Characterized by chronic inflammation and inappropriate immune responses, IBD may develop into stenosis disease, fistula phenotypes or even CRC, posing a serious management challenge. Despite many years of research, the exact pathogenesis has not been completely elucidated. Current data indicate that IBD could be accounted as the result of the complex interplay between genetic predispositions, environmental factors, and aberrant immune responses (Kaser et al., 2010; Zhang et al., 2018). Although recent technological advances have enormously facilitated the genetic research in IBD, the identified genetic factors can only explain a small proportion of overall disease variance (Ventham et al., 2013). Moreover, the great differences in disease manifestations between young and old patients cannot be explained merely by different genotypes; environmental factors should also be given due importance due to the finding that environmental changes could shape pathological gene expression through epigenetic mechanisms (Aleksandrova et al., 2017). Besides, the rapidly growing incidence and steadily increasing prevalence of IBD further impelled us to uncover the role of the genome-environment interaction in the occurrence and development of IBD (Kaplan and Ng, 2017). Epigenetic mechanisms such as DNA methylation, non-coding RNAs, histone modification, and the positioning of nucleosomes significantly contribute to the interplay between genome and environment (Ventham et al., 2013; Karatzas et al., 2014). Available evidence also supports the critical roles of epigenetic modifications in the disease susceptibility, activity, behavior, and CRC of IBD, which has provided valuable insights into the molecular basis of IBD. Moreover, it is well known that the diagnosis, differential diagnosis, disease surveillance, and treatment of IBD are difficult, and until now, there wasn’t a single solution to offer an accurate diagnosis and monitoring of IBD, and completely cure IBD on its own merits (Zhang et al., 2018). In the era of precision medicine, precision diagnosis and treatment have become an increasingly important issue in clinical practice (Li, 2018; Weissman, 2018). So, defining roles of epigenetics in IBD provides new avenues for the development of disease prediction, therapy, and monitoring. In this review, we introduce the recent genetic and epigenetic discoveries in IBD, primarily focusing on the roles of epigenetics in disease susceptibility, activity, behavior, CRC, and the potential translational applications.

## Achievements of Genetic Research in IBD

Early family and twin studies have demonstrated that genetic factors play a fundamental role in disease susceptibility of IBD. The prevalence of disease (CD or UC) among relatives of IBD patients was significantly higher than that in controls. It should be emphasized that consistent trends were noticeable. The relatives of CD patients were at higher risk of developing CD, and those of UC patients were more likely to be subjected to UC than CD (Satsangi et al., 1994). Twin studies not only suggested that the twin concordance rates were much higher in CD than in UC, but also claimed that twins with IBD represented great consistency in clinical characteristics (Satsangi et al., 1994; Halfvarson et al., 2003). Later linkage analyses and association studies further identified many susceptibility loci (*IBD1-9*) of IBD. Nucleotide binding oligomerization domain containing 2 (*NOD2*, also known as *CARD15*) gene located in the IBD1 locus was firstly demonstrated to be a risk allele of CD, and three rare SNPs (R702W, G908R and 1007fs) were the most studied (Ahmad et al., 2001; Zhang et al., 2018). It is noteworthy that, Helbig et al. (2012) found cigarette smoking to be a possible modulator of the *NOD2* mRNA expression and function, and therefore *NOD2*-smoking interaction (gene–environment interaction) might confer an increased risk to CD. Technological innovations such as Genome-wide association study (GWAS), whole exome sequencing (WES), and fine-mapping have dramatically facilitated genetic research in IBD, identifying more than 240 susceptibility loci of IBD, including TNF superfamily member 15 (*TNFSF15*), interleukin 23 receptor (*IL23R*), autophagy related 16 like 1 (*ATG16L1*), immunity related GTPase M (*IRGM*), PR/SET domain 1 (*PRDM1*), and nuclear dot protein 52 kDa (*NDP52*, also known as *CALCOCO2*) (Ellinghaus et al., 2013; Liu et al., 2015; de Lange et al., 2017). Among these risk loci, some are shared by both CD and UC, while others are specific to one subtype (CD or UC). These data indicate that genetics plays a role in the pathogenesis of both CD and UC. However, it was quite disappointing to discover that the heritability conferred by genetic predisposition is smaller than expected (also known as missing heritability). Available data indicate that the portion of heritability explained by genetic variants was only 13.1% in CD, and 8.2% in UC (Liu et al., 2015). Therefore, understanding the role of other factors such as epigenetic modifications is a vital step in uncovering the sophisticated pathogenesis of IBD.

## Epigenetic Modifications in IBD

Epigenetic modifications are defined as changes to gene structure and heritable phenotype that cannot be explained by altered DNA sequences. The classic epigenetic mechanisms include DNA methylation, histone modification, non-coding RNAs, and nucleosome positioning. In contrast, some new modifications such as RNA methylation are on the horizon (Ventham et al., 2013; Huang et al., 2019). Among these modifications, DNA methylation and non-coding RNAs are most extensively studied in IBD research.

DNA methylation is one of the chemical modifications of DNA. It is referred to the covalent addition of a methyl group to cytosines, which mostly occurs at cytosine phosphate guanine (CpG) dinucleotides, resulting in 5-methylcytosine formation (Jeltsch et al., 2018; Li et al., 2019). CpG dinucleotides occur in human genome with a low frequency of 1%, and present with nonrandom distribution (Portela and Esteller, 2010). Regions relatively clustered with CpG dinucleotides are named as CpG islands (CGIs) that range from 200bp to 5kb in length, preserve in 1–2% of the genome, and show a decreased transcriptional activity (Tang and Ho, 2007). Several studies have demonstrated aberrant changes of DNA methylation in IBD patients (Tahara et al., 2009a; Cooke et al., 2012; Kang et al., 2016; McDermott et al., 2016). Alterations in the methylation status of IBD-associated genes considerably change the transcriptional activity and expression levels of genes, thereby shaping the disease risk and progression. It is noteworthy that some DNA profiles are claimed to be common to both CD and UC, while others are demonstrated to be specific for CD or UC, which create novel and powerful motivations for disease classification and therapy. In addition, some aberrant methylated genes were initially found to be involved in IBD, and were not identified as IBD risk genes before. In this regard, it would cast new insights into the intricate pathogenesis of IBD. Non-coding RNAs are a group of RNA molecules that are not translated into proteins, including small interfering RNA (siRNA), microRNA (miRNA), PIWI-interacting RNA (piRNA), long non-coding RNA (lncRNA) and others (Gutschner and Diederichs, 2012). Numerous cellular processes such as translation, RNA splicing, gene and chromosome structure modulation, as well as DNA replication and genome defense are correlated with these non-coding RNAs (Winter et al., 2009; Gutschner and Diederichs, 2012; Dong et al., 2018). Current data indicate that non-coding RNAs, especially miRNAs, generally act in 3′ untranslated regions (3′ UTRs) and 5′ UTRs of genes, regulating gene expression at both transcriptional and post-transcriptional levels, and modifying the IBD-correlated mechanisms such as T-cell differentiation, IL23/Th17 signaling pathways, and autophagy; as a result, affecting the disease onset and progression (Wilusz et al., 2009; Winter et al., 2009; Gutschner and Diederichs, 2012; Kalla et al., 2015; Dong et al., 2018). In accordance with findings of DNA methylation, some non-coding RNAs are also differentially expressed between CD and UC. In this respect, miRNAs can serve as potential biomarkers to provide supplementary information for more precise diagnosis and management of IBD. Collectively, it is an important emerging area as epigenetic modifications play a key regulatory role in gene replication, gene expression, and chromatin remodeling. However, despite rapid progresses being made in the field, other epigenetic patterns such as histone modification and nucleosome positioning are less studied in IBD. Moreover, the functions and precise mechanisms for most of epigenetic modifications are not completely understood. Therefore, it is definitely a pressing need to devote more efforts to annotate the functions and mechanisms of epigenetic changes in IBD. Applying basic research results into reliable biomarkers and therapeutic strategies is also becoming increasingly necessary.

## Roles of Epigenetics in IBD

Epigenetic modifications are involved in numerous diseases including cancers, neurodevelopmental disorders, cardiovascular diseases, and autoimmune diseases (rheumatoid arthritis, psoriasis, and IBD). Established roles of epigenetic modifications in the pathogenesis of these diseases suggest novel targets for disease therapy. Furthermore, significant associations between epigenetic modifications and disease susceptibility, activity and behavior indicate a potential ability to diagnose and manage disease. In this paper, we introduce the roles of epigenetic modifications in IBD, with a focus on DNA methylation and miRNA profiles (**Tables 1** and **2**).

**Table 1:** Roles of DNA methylation in IBD.

Methylated markers	Methylation status (↑/↓)	Roles
**Estimation of disease susceptibility**		
THRAP2, FANCC, GBGT1, WDR8 and ITGB2	↑	CD vs. healthy controls
DOK2, TNFSF4 and VMP1	↓	CD vs. healthy controls
THRAP2, FANCC, GBGT1, WDR8, CARD9 and CDH1	↑	UC vs. healthy controls
ICAM3, DOK2, TNFSF4 and VMP1	↓	UC vs. healthy controls
GBGT1, IGFBP4 and FAM10A4	↑	CD vs. UC
IFITM1	↓	CD vs. UC
**Assessment of disease activity**		
CDH1, GDNF, SLIT2, MDR1, FMR1, GXYLT2 and RARB	↑	Active UC vs. quiescent UC
FOXA2, ROR1, NOTCH3, CDH17, PAD14, TNFSF8, EPHX1, HOXV2 and FRK	↓	Active UC vs. quiescent UC
SLIT2	↑	Active CD vs. quiescent CD, correlates with endoscopic and histological activity
Evaluation of disease behavior		
PAR2	↑	Total colitis phenotypes, steroid-dependent and refractory phenotypes of UC, and stricturing phenotypes
MDR1	↑	Total colitis phenotypes, younger onset of disease, and chronic continuous type of UC
CDH1, CDH13 and GDNF	↑	Long-standing disease course of UC
miR-1247 and CDX1	↑	Refractory UC and severe Mayo endoscopic score
RPS6KA2	↑	Stricturing/penetrating phenotypes of CD, and extensive disease of UC
**Cancer surveillance**		
RUNX3, MINT1, TGFB2, SLIT2, HS3ST2, TMEFF2, ITGA4, TFPI2, FOXE1, SYNE1 APC, CDH13, MGMT and MLH1	↑	Discriminate UC–CRC from controls
COX-2	↓	Discriminates UC–CRC from controls
miR-137	↑	Discriminates dysplasia and UC-CRC from controls
BMP3, vimentin, EYA4 and NDRG4	↑	Discriminate neoplasia and CRC from controls

**Table 2:** Roles of miRNAs in IBD.

miRNAs	Expression levels (↑/↓)	Roles
**Estimation of disease susceptibility**		
miRs-3180-3p, miRplus-E1035 and miRplus-F1159	↑	Active UC vs. active CD and healthy controls
miR-20b, miR-98, miR-125b-1* and let-7e*	↑	Active UC vs. inactive UC, active CD, inactive CD, and healthy controls
miRs-103-2*, miR-362-3p, miR-532-3p, miR-20b, miR-98, miR-125b-1* and let-7e*	↑	UC vs. healthy controls
miR-340* and miR-484	↑	CD vs. healthy controls
**Assessment of disease activity**		
miR-16, miR-21, miR-24, miR-126, miR-203, miR-28-5p, miR-151-5p, miR-199a-5p, miR-340*, miRplus-E1271 and miR-595	↑	Active UC vs. quiescent UC
miR-200b and miR-124	↓	Active UC vs. quiescent UC
miR-199a-5p, miR-362-3p, miR-532-3p, miRplus-E1271, miR-877 and miR-595	↑	Active CD vs. quiescent CD
miRplus-F1065	↓	Active CD vs. quiescent CD
**Evaluation of disease behavior**		
miR-23b, miR-106 and miR-191	↑	Colonic CD
miR-19b and miR-629	↓	Colonic CD
miR-16, miR-21, miR-223 and miR-594	↑	Ileal CD
miR-29a, miR-29b, miR-29c, miR-19a-3p and miR-19b-3p	↓	Stricturing phenotypes of CD
miR-31-5p	↑	Stricturing and/or penetrating phenotypes
miR-196b-5p and miR-149-5p	↓	Stricturing and/or penetrating phenotypes
**Cancer surveillance**		
miR-31 and miR-224	↑	Discriminate dysplasia and CRC from controls, as well as IBD-associated CRC from sporadic CRC
miR-143 and miR-145	↓	Correlate with neoplastic progression of IBD
miR-21	↑	Discriminates dysplasia and CRC from controls
miR-155	↑	Correlates with neoplastic progression of IBD
miR-26b	↑	Discriminates UC–CRC from controls
miR-15b, miR-17, miR-26b and miR-145	↑	Discriminate CRC from controls

### Estimation of Disease Susceptibility

It is well established that traditional diagnosis and differential diagnosis of IBD are based on comprehensive analysis of clinical characteristics, laboratory parameters, endoscopy, imaging features, and histologic examinations. Other emerging surrogates such as genetic, serological, histologic, and fecal markers have also showed an important potential in disease diagnosis and classification. Although with these methods, some patients are still diagnosed with “IBD-unclassified” or “indeterminate colitis” (Satsangi et al., 2006). Therefore, identification of more diagnostic markers for IBD is of paramount importance. Epigenetic modifications such as DNA methylation and miRNAs are attractive biomarkers for diagnosis at a molecular level. A large number of studies have demonstrated the strength of sensitivity, specificity, and accuracy in the diagnosis of IBD.

Cooke et al. (2012) convincingly claimed that IBD cases displayed different mucosal methylation changes (*THRAP2*, *FANCC*, *GBGT1*, *DOK2* and *TNFSF4*) in comparison to healthy controls. Besides, they also found a significant difference in methylation landscape between CD and UC patients. For example, CD patients showed hypermethylated *GBGT1*, *IGFBP4*, *FAM10A4* and hypomethylated *IFITM1* when compared with UC patients, which provides a possibility for discriminating IBD from controls, and CD from UC. Subsequently, Adams et al. (2014) suggested that CD patients displayed different circulating leukocyte methylation profiles in comparison to healthy controls. They identified 65 probes and 19 differentially methylated regions (DMRs) in pediatric patients with CD, and developed models for each possible combination of two probes to discriminate CD and healthy controls with AUCs ranging from 0.79 to 0.98 (mean value of 0.93). However, no direct comparison between CD and UC has been made in their study. It is worth noting that most methylation changes occurred in proximity to GWAS risk loci. These results accord with a similar finding by Cooke et al. (Cooke et al., 2012). They demonstrated that many identified GWAS risk genes (*CARD9*, *CDH1*, *ICAM3* etc.) presented different methylation status between IBD patients (CD and UC) and healthy controls, suggesting a possibility of mechanistic interactions between the epigenetic and genetic signals. Existing data exhibited that referred SNPs could be located in CGIs, disrupt CpG sites, and therefore interfere CGI methylation states (Cooke et al., 2012). Meanwhile, methylation alterations in or in proximity to the transcription start site and the promoter region of susceptibility genes also exert great influence on gene transcription (Adams et al., 2014). This indicated that genetic risk loci might mediate effects on disease susceptibility through DNA methylation. In 2016, an epigenome-wide association study (EWAS) of 240 newly-diagnosed adult patients with IBD (CD and UC) and 190 controls successfully identified four DMRs (*VMP1*, *ITGB2*, *WDR8* and *CDC42BPB*) in CD versus controls, and two DMRs (*VMP1* and *WDR8*) in UC in comparison with controls, which paralleled the genomic findings that CD and UC not only have their own specific susceptibility loci, but also share overlapping risk loci to some extent. Furthermore, Ventham et al. (2016) also created a diagnostic model of 19 methylation probes that could distinguish CD from UC with a favorable sensitivity of 1 and acceptable accuracy of 0.719. Another 30-probe panel could differentiate IBD patients from controls with a sensitivity, a specificity, and an AUC of 0.812, 0.847, and 0.898, respectively (Ventham et al., 2016). Recently, a British research team has revealed distinct gut segment-specific DNA methylation patterns of intestinal epithelial cells (IECs) between pediatric IBD patients and healthy controls. Their data indicated that disease-specific DNA methylation profiles of IECs (ascending colon) could accurately separate IBD patients from healthy controls with a sensitivity of 75% and a specificity of 100%. Moreover, another ileal methylation signatures were capable of distinguishing CD from UC with a precision of 77% and an AUC of 0.92 (sensitivity of 57%, specificity of 100%) (Howell et al., 2018). Such a high degree of diagnostic value suggests its potential utility in clinical settings. Successful application of DNA methylation markers in cancer detection and surveillance has paved new ways for IBD research. Compared to genetic biomarkers, DNA methylation incorporates cumulative or specific environmental experience (such as smoking and diet) and the influence of age. Besides, current methylation detection encompasses panels of multiple methylation markers rather than a single marker, showing its superiority in sensitivity and specificity (Laird, 2003). Furthermore, DNA methylation biomarkers are stable in the bloodstream, tissues and even in stool, making it convenient to be preserved and detected (Johnson et al., 2016). Moreover, methylation assays for individual DNA methylation surrogate tend to be universal, which is similar to genetic markers (Laird, 2003). However, there are still some factors limiting the routine clinical application. Firstly, as is well known, DNA methylation signatures are cell-specific. Different sampling sites may exhibit a marked difference in DNA methylation profiles due to different types of cells located in these sites (Cooke et al., 2012). Secondly, substantial (45%) overlap of differentially methylated positions (DMPs) between UC and CD might bring additional hurdles with regard to discriminating between them (McDermott et al., 2016). Thirdly, limitations of technologies applied in DNA methylation analyses significantly restrict clinical translation. The bisulphite-based approaches are still the leading methods used in this field. High quality samples and DNA sequence bias are important and serious challenges for a long time. Although whole genome bisulfite sequencing (WGBS) has displayed advantages in sample requirement, high coverage, and less DNA sequence bias, additional efforts are still in pressing need in order to resolve difficulties in PCR polymerase and bisulfite conversion (Raine et al., 2017). Fourthly, expensive testing costs need to be taken into consideration, which can add to the financial burden and thus, decrease patient acceptance. DNA methylation markers are indeed a powerful and promising tool to make a diagnosis of IBD. However, more studies are warranted prior to their clinical application.

MicroRNAs (miRNAs) are a group of non-coding RNAs with a length of about 22 nucleotides, mediating RNA silencing and gene expression regulation at a post-transcriptional level (Fisher, 2015). Accumulated evidence has showed its critical contribution to disease onset and progression of IBD, which supports possibilities of exploring roles of miRNA markers in diagnosis and differential diagnosis. miRNA expression patterns significantly differ between IBD patients and healthy controls, CD patients and UC patients, as well as between patients in remission and those in active states. Wu et al. (Wu et al., 2011) identified a panel of three peripheral blood miRNAs (miRs-3180-3p, miRplus-E1035 and miRplus-F1159) that were differentially expressed in active UC patients and healthy controls, and they also could distinguish active CD patients from UC patients. In the same study, specific miRNA expression panels of CD and UC have also been reported. Patients with UC displayed higher levels of miRs-103-2*, miR-362-3p, and miR-532-3p compared with healthy controls, irrespective of whether they were in remission or in active status. However, CD patients always displayed increased levels of miR-340* in peripheral blood. A further study has identified four specific miRNA surrogates (miR-20b, miR-98, miR-125b-1*, and let-7e*) in colonic mucosa of UC patients and claimed that they were differentially up-regulated by more than 5-fold in active UC in comparison to inactive UC, active CD, inactive CD, and healthy controls, driving its continuous development in IBD discrimination (Coskun et al., 2013). Zahm et al. (2011) tested the diagnostic ability of 11 serum miRNA markers in pediatric patients with CD, and found that these miRNA surrogates could accurately differentiate CD patients from controls with sensitivities higher than 80%. Among these identified miRNAs, miR-484 outstripped other miRNAs and promising markers, including C-reactive protein (CRP), anti-*Saccharomyces cerevisiae* antibody (ASCA) IgG, erythrocyte sedimentation rate (ESR) and albumin, with an AUC of 0.917, a sensitivity of 82.61%, and a specificity of 84.38%, respectively. However, the discriminative power of these CD-associated miRNAs in distinguishing CD from UC, CD from irritable bowel syndrome (IBS), and CD from celiac disease is unknown. More studies are warranted to elucidate the discriminative capacity with regard to these differential diagnoses. Even though peripheral blood and colon mucosa miRNA markers play a pivotal role in disease diagnosis, limitations including invasiveness, inflexibility, and time consumption make them unacceptable for patients. Saliva miRNA markers might overcome these shortcomings and provide additional diagnostic information. Different saliva miRNA expression signatures between IBD cases and healthy controls may help physicians in disease diagnosis and classification (Schaefer et al., 2015). In order to improve diagnostic accuracy, extended panels may be more helpful. A study of 76 IBD (CD and UC) patients and 38 healthy controls has established classification models comprising of various miRNAs (miR-34b-3p, miR-377-3p, miR-484, miR-574-5p etc.), which could discriminate IBD from healthy controls, and CD from UC, with increased AUCs of 0.89 to 0.98, and low classification error rates of 3.3% and 3.1%, respectively (Chamaillard et al., 2015). More importantly, some studies have observed a considerable overlap of miRNA signatures between IBD and other immune diseases (systemic lupus erythematosus, rheumatoid arthritis, asthma etc.), paralleling the genetic overlap between IBD and other immune diseases, which suggested some shared pathways among them; thereby offering a possibility of knowledge innovation in diagnosis and targeted treatment of IBD (Lees et al., 2011; Wu et al., 2011; Clark et al., 2012). In addition, it is important to note that clear differences of miRNA expression signatures have also been observed in different studies, that is to say, increased levels of miRNAs that were identified in one study otherwise showed a decreased expression in another study, or altered miRNAs couldn’t be validated in other studies, which made it somewhat difficult for physicians to make an accurate diagnosis. In addition to different miRNA microarray platforms and sample sizes, other influencing factors such as different sample resources (colon tissues, peripheral blood, stool, saliva etc.) and inconsistent fold change criteria, as well as different therapeutic regimens, disease states (active or quiescent), and disease duration may also account for it (Coskun et al., 2013; Kalla et al., 2015; Schaefer et al., 2015). Thus, these reported miRNA markers are needed to be validated in large-scale, independent, clinically well-matched cohorts.

As for histone modifications and nucleosome positioning, definite evidence is still lacking for the contributions in diagnosis and differential diagnosis of IBD. Available evidence demonstrated complex networks between DNA methylation, miRNAs, histone modifications and nucleosome positioning. (Wang et al., 2013). So, determined DNA methylation or miRNA markers may affect disease susceptibility through histone modifications or nucleosome positioning at some levels. Therefore, further studies are warranted to clarify the detailed interactions, functional pathways and transcription regulation amongst these epigenetic modifications.

Diagnosis and differential diagnosis of IBD are definitely a major clinical challenge. Collection of additional evidence might help achieve a higher diagnostic accuracy of IBD. Emerging molecular markers such as DNA methylation and miRNA markers, along with other surrogates such as *NOD2*, ASCA, antineutrophil cytoplasmic antibody (ANCA), fecal calprotectin (FC) and fecal lactoferrin (FL), have exhibited certain advantages over other classic surrogates with regard to the sensitivity, specificity and accuracy (Zhang et al., 2018). A pooled analysis of different-class markers ensures a more precise diagnosis, but cost–effectiveness ratio should also be taken into account. Although most of these emerging molecular markers have not been recommended in any guidelines, and are not usually generalized in routine diagnosis, they indeed provide some useful diagnostic information for doctors. Considering that some results were obtained in small sample studies, verification in larger, well-designed, and prospective studies has become increasingly important.

### Assessment of Disease Activity

The natural course of IBD is characterized by relapse-remission. A population-based study from Copenhagen has delineated that approximately 18% patients could experience an indolent course, with 57% undergoing moderate activity (no less than two relapses within the first five years, but less than every year), and 25% having aggressive disease (disease relapses every year) during the first 5 years after diagnosis of CD (Jess et al., 2007). The corresponding percentages of UC of indolent, moderate, and aggressive disease course were 13%, 74% and 13%, respectively (Jess et al., 2007). IBD patients with earlier recurrence are at higher risk of relapsing during following years than those with later relapse (Magro et al., 2017). In routine clinical work, patients with relapse are recommended to get microbiological examination of stool, serological tests such as ESR and CRP, and even sigmoidoscopy or colonoscopy, aiming to exclude specific infections and assess disease activity. However, classic markers are not always parallel to disease activity. Some patients with mild or moderate disease activity may display normal serological parameters (Magro et al., 2017). Additionally, other diseases such as infectious enteritis and intestinal tuberculosis can also result in abnormal levels of ESR and CRP, making them unspecific for IBD (Zhang et al., 2018). Even though endoscopy together with histological analysis is recognized as the gold standard for the assessment of disease activity, it is unreasonable to prescribe endoscopy for patients once the disease flares. In recent years, novel epigenetic markers are claimed to be independently correlated with disease activity, and be of practical significance in the assessment of disease activity.

Saito et al. (2011) analyzed colonic methylation levels of UC patients and found that inflamed mucosa exhibited markedly higher methylation status of cadherin 1 (*CDH1*) and glial cell derived neurotrophic factor (*GDNF*) loci compared with quiescent mucosa. Recently, Barnicle et al. (2017). compared the DNA methylation patterns in inflamed and non-inflamed tissues of UC patients, and successfully found four differentially methylated and expressed genes (*ROR1, GXYLT2, RARB*, and *FOXA2*) that were involved in the regulation of Wnt signaling and cell development. A further study of 38 IBD patients (29 UC and 9 CD) revealed a significant correlation between slit guidance ligand 2 (*SLIT2*) methylation and endoscopic and histological activity (Lobatón, 2014). It should be pointed out that altered methylation status was also correlated with changed endoscopic activity in the longitudinal study. *SLIT2* methylation status tended to be elevated in patients who shifted from remission to active states. In addition, a large-scale systematic review of 16 studies further identified 25 differentially methylated inflammatory genes between UC patients and controls (Gould et al., 2016). Among these genes, methylation status of multidrug resistance 1 (*MDR1*), fragile X mental retardation 1 (*FMR1*), *CDH1* and *GDNF* gene was elevated, while methylation status of *NOTCH3, CDH17, PAD14, TNFSF8, EPHX1, HOXV2, FRK* etc. was decreased in inflamed mucosa in comparison with quiescent mucosa, indicating that histologic methylation profiles can serve as valuable surrogates to evaluate the disease activity of IBD. Associations between serum methylation signatures and disease activity have also been corroborated by several other studies (Gould et al., 2016). However, a recent genome-wide DNA methylation study has drawn a contrary conclusion that peripheral blood DNA methylation was not significantly different between active and inactive disease states (McDermott et al., 2016). Considering great heterogeneity of disease locations, disease duration, disease behaviors, degrees of disease activity, and drug use might affect the epigenetic changes, large scale, well-matched, prospective studies are needed to further verify the relationships between them. It must be stressed that most methylated loci have been confirmed to be IBD susceptibility loci by GWAS, while some methylated loci were firstly identified in these epigenetic studies, and were demonstrated to be involved previously unknown signaling pathways. In this sense, this offered a possibility of unveiling new pathogenic mechanisms of IBD and developing new targets for treatment (Saito et al., 2011; Lin et al., 2012; McDermott et al., 2016). Given that blood collection is more accessible and less invasive than biopsy, some studies compared the DNA methylation changes between peripheral blood and intestinal tissues, and suggested that methylation profiles in peripheral blood could reflect DNA methylation patterns in intestinal tissues (Gould et al., 2016; McDermott et al., 2016). Thus, identification of serum methylation signatures may be more acceptable in the assessment of disease activity. However, there is still a lack of studies directly comparing the diagnostic accuracy of methylation markers with other classic and emerging markers, additional efforts should be made to fill this gap.

miRNAs were firstly reported to be of value in the evaluation of disease activity of IBD in 2008 (Wu et al., 2008). Expression levels of miR-16, miR-21, miR-24, miR-126 and miR-203 were increased in active UC tissues in comparison with quiescent UC tissues. In contrast, miR-200b displayed a lower expression concentration in active UC tissues than in inactive ones (Wu et al., 2008). Among these differentially expressed miRNAs, miR-21 showed the highest fold change of 3.7 between active and inactive disease states. It should be emphasized that no difference has been found in the expression levels of the active UC-associated miRNAs between CD patients and controls. A later study also confirmed that peripheral blood miRNAs could distinguish active IBD from quiescent IBD (Wu et al., 2011). Their data demonstrated that active CD patients displayed an increased expression level of miR-199a-5p, miR-362-3p, miR-532-3p and miRplus-E1271 as well as a decreased level of miRplus-F1065, compared with CD patients in remission. Similarly, as for UC patients, miR-28-5p, miR-151-5p, miR-199a-5p, miR-340* and miRplus-E1271 were elevated in active ones but not in inactive ones. Moreover, miRs-3180-3p, miRplus-E1035 and miRplus-F1159 were demonstrated to be differentially expressed in the active UC patients vs active CD patients, which supported the hypothesis that the two subtypes of IBD were implicated in different pathogenic mechanisms. Additional serum or tissue miRNA markers such as miR-124, miR-877, miR-595 etc. are also claimed to be instrumental in discriminating active IBD from inactive IBD (Iborra et al., 2013; Koukos et al., 2013; Krissansen et al., 2015). In 2016, a spearman correlation analysis indicated that circulating miR-223 was not only correlated with ESR and hs-CRP, but also correlated with clinical activity index including Crohn’s Disease Activity Index (CDAI), Simplified Endoscopic Score for Crohn’s Disease (SES-CD), Mayo score, and Ulcerative Colitis Endoscopic Index of Severity (UCEIS) (Wang et al., 2016). However, little is known about the definite predictive values (sensitivity, specificity and AUC) of these miRNAs in detection of disease activity in IBD. Moreover, there is still a lack of evidence about comparative advantages of miRNA markers when compared with other accurate markers such as serum calprotectin (SC), FC and FL. Another important issue that should be stressed is that whether serum expression profiles of IBD-associated miRNAs can reflect miRNA expression patterns in intestinal tissues. Contrary results have been found in some studies (Archanioti et al., 2011; Iborra et al., 2013; Zhang et al., 2018). So, larger comparison studies of paired serum and mucosal tissues are warranted. This further merits additional investigation to see if the combined analysis of serum and histologic miRNA profiles will ensure a more accurate assessment of disease activity.

### Evaluation of Disease Behavior

IBD is a heterogeneous entity with distinct disease locations, age of onset, phenotypes, and severity. A majority of patients experience great changes of disease behaviors throughout the disease course. For example, some CD patients with inflammatory phenotypes may convert into stricturing or penetrating phenotypes, and UC patients manifesting proctitis will develop into extensive colitis as the disease progresses. Some convincing evidence suggests that early age onset, extensive disease, the presence of perianal disease, and stricturing or penetrating subtypes are risk factors of progressive course and poor prognosis (Gomollon et al., 2017; Magro et al., 2017). Screening patients with a less favorable course in the early stage of disease is highly recommended. So, it is of paramount importance to identify markers that can help physicians evaluate disease behavior in clinical practice.

Tahara et al. (2009b) firstly demonstrated that protease-activated receptor 2 (*PAR2*) methylation status was independently associated with various clinical disease behaviors in a study of 84 UC patients. Their data indicated that methylation levels of *PAR2* tended to be higher in patients with total colitis in comparison to those with rectal colitis, and increased methylation levels were also correlated with steroid-dependent and steroid-refractory phenotypes. In the same year, Christerson et al. (2009) suggested that PAR2 activation could potentiate intestinal myofibroblast proliferation and stricture formation in patients with CD. Considering that *PAR2* is widely implicated in the regulation of inflammatory responses, cell growth, and stricture formation in IBD, PAR2 methylation markers may serve as a valuable tool in the assessment of disease behavior (Christerson et al., 2009; Tahara et al., 2009b). In the same year, Tahara’s research team further identified the putative roles of *MDR1* methylation signatures in UC patients. They suggested that increased methylation levels of *MDR1* gene were not only associated with total colitis phenotypes, but also correlated with younger onset of disease (≤20 years) and chronic continuous types (Tahara et al., 2009a). Available evidence has demonstrated a close association between *MDR1* dysfunction and impaired intestinal epithelial barrier in UC. Moreover, those patients who had progressive disease course were more likely to present severely damaged intestinal epithelial barrier (Schwab et al., 2003; Tahara et al., 2009a). From this point, *MDR1* methylation surrogates may be of important value and significance in evaluation of disease course. Further evidence has indicated that *CDH1*, *CDH13* and *GDNF* methylation occurred more frequently in UC patients with long-standing disease course, and higher methylation status of miR-1247 and caudal type homeobox 1 (*CDX1*) could serve as a predictor of refractory UC and severe Mayo endoscopic score (Saito et al., 2011; Schneider-Stock et al., 2014; Gould et al., 2016). In addition, hypomethylation of ribosomal protein S6 kinase A2 (*RPS6KA2*) has also been identified as a diagnostic aid in the prediction of complicated disease behavior (stricturing/penetrating disease) of CD and extensive disease of UC (Ventham et al., 2016). *RPS6KA2* is a ribosomal kinase that is responsible for the modulation of cell growth, motility and proliferation, as well as the regulation of PI3K/Akt/mTor pathway and autophagy. The latter has been proven to be one of the most important pathogenesis of CD in recent years. Previous studies have declared that gene expression is characterized by region-specificity in intestine (Bates et al., 2002). Given that DNA methylation can regulate gene expression at a post-transcriptional level, a significant difference of DNA methylation status in different segments of intestine may help explain the underlying molecular basis. Moreover, close associations between methylation status and certain disease behaviors highlight the exciting potential of using methylation markers in the assessment and prediction of disease behaviors. However, comparative studies are still in need to assess the exact predictive value in IBD, and extended panels of different molecular markers are also required to improve the accuracy of prediction.

The fact that the expression of miRNAs in intestine is region-specific, provides a basis for studying the specific miRNA expression patterns in IBD patients with different disease locations. Wu et al. (2010) have successfully identified three specifically upregulated miRNAs (miR-23b, miR-106 and miR-191) and two down-regulated miRNAs (miR-19b and miR-629) in tissues from colonic CD, and four miRNAs (miR-16, miR-21, miR-223, and miR-594) with increased expression in tissues from ileal CD, offering a possibility of using miRNA biomarkers to discriminate different subtypes of CD. Moreover, a British study highly suggested that the expression levels of miR-29 family were in correlation with stricturing phenotypes in CD patients (Nijhuis et al., 2014). They conducted a comparative study of mucosa overlying a stricture and paired non-stricturing samples in CD patients, and claimed that expression levels of miR-29a, miR-29b and miR-29c were significantly down-regulated in mucosa overlying a stricture compared with the other. Similarly, in the serum, there is also a great reduction of expression levels of miR-29a in patients with stricturing phenotypes in comparison to those manifesting inflammatory phenotypes. This data was in accordance with previous findings that a decreased level of miR-29 family is a hallmark of cardiac, hepatic, pulmonary, and renal fibrosis, suggesting its significant contribution in tissue fibrosis (Nijhuis et al., 2014; Lewis et al., 2015b). A later study of 106 patients with CD further suggested that reduced serum expression levels of miR-19-3p (miR-19a-3p and miR-19b-3p) were independently associated with stricturing phenotypes (Lewis et al., 2015a). More importantly, further evidence showed that decreased serum miR-19-3p levels antedated the development of stricture, and remained low in patients with resected strictures. In addition, Lewis et al. (2015a) compared the predictive value of miR-19-3p, disease duration and ileal disease in discriminating stricturing from non-stricturing subtypes, and found that disease duration outperformed the other indicators with an AUC of 0.76, followed by miR-19-3p (AUC = 0.67) and ileal disease (AUC = 0.58). Combined analysis of the three predictors would make the classification efficiency increase markedly, with an AUC of 0.81. Additionally, some other miRNA markers such as miR-31-5p, miR-196b-5p, miR-149-5p etc. have also been confirmed in association with stricturing and/or penetrating phenotypes (Peck et al., 2015). Distinct expression patterns of miRNAs are of high value as a diagnostic and predictive tool in classifying different disease behaviors of IBD patients. Current diagnostic modalities displayed a limited value in discriminating inflammatory stricture from fibrotic stricture, while miR-29 family showed a great potential in identifying stricturing subtypes secondary to fibrosis (Nijhuis et al., 2014). Exploration of additional miRNA markers capable of classifying inflammatory and fibrotic stricture is in an unmet need, as this could guide clinicians in implementing individualized treatment (drug therapy, endoscopic balloon dilation or surgical intervention). In addition, establishing a standardized miRNA processing protocol is in dire need, considering that different RNA isolation methods and miRNA microarray platforms greatly influence the experimental results (Lewis et al., 2015a; Lewis et al., 2015b). Furthermore, functional significance and targeted sites of miRNA markers also deserve in-depth investigation in order to unveil the comprehensive molecular basis of IBD and develop miRNA-based therapeutics.

In the era of precision medicine, physicians are advised to perform risk stratification firstly according to the clinical characteristics, endoscopic findings, and imaging features, as well as molecular markers, and then select the most suitable treatment for individual patient based on risk stratification. Epigenetic patterns indeed provide some important clues for disease risk. Based on the risk analysis, patients can be divided into two groups including high risk group and low risk group, and the two different groups are supposed to receive different treatment regimens. The European Crohn’s and Colitis Organization (ECCO) consensus recommends that patients with poor prognosis and progressive disease course better receive early and progressive therapy (immunomodulator or biological agents) and if possible, a combined treatment of immunosuppressant and biological agents. For patients with mild course, an accelerated step-up approach is recommended, which markedly decreases the unnecessary expenses and the risk of severe adverse events (Gomollon et al., 2017; Harbord et al., 2017). However, epigenetic markers have not been included in any guidelines for IBD treatment, suggesting many areas need to be improved. In addition, even we can choose de-escalation or escalation therapy according to risk stratification. The challenge remains to select the most suitable drugs for each individual amongst a variety of drugs, given that different patients show significantly different drug metabolism rates and response rates to therapy. Genetic markers such as thiopurine S-methyltransferase (*TPMT*), nucleoside diphosphate-linked moiety X-type motif 15 (*NUDT15*), and inosine triphosphate pyrophosphatase (*ITPA*) variant loci that implicate drug metabolism have been shown to be of great value in predicting therapeutic efficacy and adverse drug reactions of thiopurines (Lucafo et al., 2018b). With the wide use of biologics (infliximab, adalimumab, vedolizumab and ustekinumab) in clinic, emerging genetic markers (*IL23R, TNFAIP3 and TNFRSF1A*) and other serologic, histologic, and fecal surrogates (CRP, ANCA, membrane-bound TNF, TNF-α, FC etc.) represent as exciting indicators for the prediction of response rates to biologics (Zhang et al., 2018). As for epigenetic profiles, available data has shown that miR-499 was associated with steroid dependence, and a high level of lncRNA growth arrest-specific 5 (GAS5) was claimed to be correlated with poor steroid response (Okubo et al., 2011; Lucafo et al., 2018a). Serum let-7d and let-7e have been found to be candidate biomarkers for the prediction of treatment response to infliximab in CD patients (Fujioka et al., 2014). Moreover, DNA methylation patterns in IECs of pediatric IBD patients were also linked with the requirement of biologics and time to third treatment escalation (Howell et al., 2018). However, definite predictive values of these epigenetic markers are still absent, which limits the clinical application to some extent. Exploring the sensitivity, specificity, predictive accuracy in other prospective and independent cohorts is of utmost importance. Considering that there are still only a limited number of studies demonstrating the roles of epigenetics in the assessment and prediction of therapeutic response, and the selection of therapeutic methods, especially in the field of the immunosuppressant and biologics, additional studies are needed to replicate these findings and find more accurate epigenetic biomarkers.

### Cancer Surveillance

IBD is a kind of long-lasting inflammatory disease with an increased risk of developing CRC, especially for patients with UC. Recent studies have shown that the cumulative risk of CRC is approximately 1.6% during fourteen-year follow-up, and UC increases the risk of CRC 2.4-fold in comparison with the normal population (Jess et al., 2012). Additionally, CRC risk increases over time as it is 8% at 20 years and 18% at 30 years after UC diagnosis (Eaden et al., 2001). Even though CRC in IBD merely accounts for a small portion (1–2%) of CRC cases in the general population, it contributes to 15% of all-causes mortality of IBD patients (Breynaert et al., 2008). Therefore, early detection and close surveillance of CRC in IBD patients are of paramount importance. Previous studies have demonstrated positive correlations between CRC and young age at diagnosis, long disease duration, extensive colitis, male, primary sclerosing cholangitis and a family history of CRC (Jess et al., 2012; Azuara et al., 2013; Luo and Zhang, 2017; Zhen et al., 2018). Serum carcinoembryonic antigen (CEA) testing and fecal occult blood testing (FOB) are the most frequently used noninvasive means of detecting CRC (Ma et al., 2019). However, these detecting means have been claimed to be less efficient with unfavorable sensitivity and specificity. Exploring robust biomarkers has become an urgent need. Emerging biomarkers such as DNA methylation and miRNAs have showed their great potential in detection and surveillance of CRC.

It is well known that DNA methylation modifications occur early in neoplasia and can work as promising early-detection indicators of carcinogenesis. In 2010, Garrity-Park et al. (2010) assessed the methylation status of ten potential genes in intestinal biopsies, and revealed significant associations between runt related transcription factor 3 (*RUNX3*), *MINT1* (also known as *APBA1*) and *COX-2* methylation and UC–CRC (OR=12.6, 9.0 and 0.2, respectively). It is noteworthy that the concurrent presence of *RUNX3*/*MINT1* methylation and COX-2 unmethylation could substantially increase the possibility of UC-CRC (OR = 61.2 and 17.6, respectively). Two years later, Azuara et al. (2013) reported that the methylation status of transforming growth factor beta 2 (*TGFB2*), *SLIT2*, heparan sulfate-glucosamine 3-sulfotransferase 2 (*HS3ST2*), and transmembrane protein with EGF like and two follistatin like domains 2 (*TMEFF2*) in colorectal biopsies could be potential surrogates for an early diagnosis of colorectal dysplasia or CRC in high-risk patients with IBD. Methylation markers of *ITGA4*, *TFPI2*, *FOXE1*, *SYNE1*, *APC*, *CDH13*, *MGMT* and *MLH1* have also proven to be high-performance screening tools for estimating individual risk for CRC or colorectal neoplasia in IBD patients (Papadia et al., 2014; Gerecke et al., 2015; Scarpa et al., 2016). A recent study by Scarpa et al. (2016) clearly identified that any two or more methylated genes (*APC*, *CDH13*, *MGMT*, *MLH1* and *RUNX3*) in the non-neoplastic mucosa could predict CRC with a sensitivity of 57.1% and a specificity of 93.1%. Such a high specificity made these methylation markers to be an ideal rule-in test to detect CRC. In addition to DNA methylation markers, miRNA methylation patterns are also helpful in detection of CRC. A large study of 238 UC patients showed that methylation of miR-137 could distinguish UC patients with dysplasia or cancer from those without neoplasia with an AUC of 0.77, and miR-1, miR-9, miR-124, miR-137 and miR-34B/C work together could accurately quantify the risk for CRC, dysplasia and neoplasia with good AUC (Toiyama et al., 2017). Considering that low-grade dysplasia (LGD) is more closely associated with UC than with CRC, and LGD does not always progress to CRC, Garrity-Park et al. (2016) extended the scope of research to UC patients with LGD, and demonstrated critical roles of *MINT1* and *RUNX3* in the progression from LGD to CRC. In the same study, researchers also established a predictive model that comprised demographic, clinical, genetic, and epigenetic indicators for detection of synchronous neoplasm, which performed better than any other traditional and experimental model with an AUC of 0.92, a sensitivity of 82.8%, a specificity of 91.2%, a PPV of 95.1% and a NPV of 72.1% (Garrity-Park et al., 2016). In addition to histological methylation markers, methylation modifications in stool are also receiving attention. Kisiel et al. (2013) tested the exfoliated DNA markers in 50 IBD patients. Fecal BMP3, vimentin, EYA4, and NDRG4 methylation markers could accurately compartmentalize CRC from controls with an AUC of 0.97, 0.97, 0.95 and 0.85, respectively. At 89% specificity, methylation BMP3 in combination with methylation NDRG4 could diagnose 100% (9/9) of CRC and 80% (8/10) of dysplasia. A later study further confirmed the predictive ability of methylated BMP3 to detect colorectal neoplasia even in small IBD lesions (Johnson et al., 2016). All the above data clearly highlight the exciting potential of methylation markers in CRC detection and surveillance. Although colonoscopy with biopsy has been proven to be the gold standard for diagnosis and monitoring of CRC or colorectal neoplasia, it is a costly, time-consuming and invasive method. Moreover, its interpretation is subject to high interobserver variability. Methylation markers do provide adjuvant and valuable messages for adjustment of surveillance interval, and formulation of an individualized treatment plan in IBD patients at different risk. Stool and saliva DNA testing, as appealing non-invasive tests, improve the patient compliance in disease monitoring. However, sample size in some studies was quite small, which limited its argumentative strength and diagnostic efficacy. Moreover, some studies neglected the influence of intestinal inflammation and neoplasia on the levels of DNA, which consequently affected the levels of methylation DNA (Johnson et al., 2016). Additionally, the morbidity of CRC exhibited great ethnic differences. Larger studies of different races are also required. It is important to stress that IBD-associated and sporadic CRC patients showed a great difference in clinical features, histopathologic characteristics, and epigenetic changes (Garrity-Park et al., 2016). Many methylation markers including *SEPT9*, *TWIST1*, *TAC1*, *IGFBP3*, *EYA4* and *SST* have been claimed to be useful in the diagnosis and surveillance of sporadic CRC, while little is known about their roles in carcinogenesis of IBD (Kisiel et al., 2013; Ma et al., 2019). Therefore, prospective studies are desperately warranted to corroborate effects of those markers in IBD-associated CRC.

Insights from miRNA research have led to salient changes in our knowledge of biological processes of CRC in IBD patients. Aberrant expression profiles of miRNAs have been claimed to be associated with IBD-associated CRC. In 2011, a preliminary study identified significant differences of miRNA expression patterns between IBD-dysplasia tissues and inflamed colonic tissues, with 22 miRNAs increased and 10 miRNAs decreased in dysplastic tissues (Olaru et al., 2011). They surprisingly found miR-31 represented a stepwise increase in the progression from normal to chronic inflammation to neoplasia, with the highest levels in CRC, which indicated its potential for an early detection of dysplasia or CRC. In addition, a marked difference of miR-31 between IBD-associated CRC and sporadic CRC made it a favorable biomarker in discriminating between them. A later study also demonstrated the successive increase of miR-224 levels at each stage of IBD progression, and its excellent performance in distinguishing IBD-cancers from non-cancers (Olaru et al., 2013). Subsequent lines of evidence indicated that miR-143, miR-145, miR-21 and miR-155 were ancillary biomarkers in the diagnosis and surveillance of IBD-associated carcinogenesis (Pekow et al., 2012; Ludwig et al., 2013; Wan et al., 2016). Relying on a single marker to detect CRC is not appropriate, establishing panels embodying different-class markers may further improve diagnostic accuracy. Benderska et al. (2015) have proven that a combined evaluation of ki-67 and miR-26b expression profiles could accurately detect 93% UC-associated colonic carcinoma. Its application in classifying different stages of CRC has also been confirmed. Recently, a Chinese research team developed a blood-based diagnostic model comprising of five circulating miRNA markers (miR-15b, miR-17, miR-21, miR-26b, and miR-145) and CEA, which could correctly diagnose CRC with an AUC of 0.85, followed by CEA of 0.793, and five-miRNA panel of 0.681 (Pan et al., 2017). However, due to the small sample size of this study, the feasibility of this diagnostic model has to be extensively studied in a larger cohort. miRNA surrogates are detectable, stable and quantifiable, with a high diagnostic and surveillance performance in discriminating CRC from controls. In this regard, miRNA biomarkers are of high clinical significance. However, many miRNA markers are not specific to CRC. Aberrant expression patterns identified in CRC are also present in other diseases. Additionally, different miRNA microarray platforms and cell types are also needed to be considered. Although the development of CRC diagnosis and monitoring is progressing at a fast pace, detection and surveillance of CRC remains challenging. Identifying more reliable markers, and establishing more robust diagnostic and surveillance models are becoming increasingly necessary. Elaborating on the roles of miRNAs in the pathogenesis and prognosis of CRC could further enhance our understanding of CRC, ultimately improve the survival quality and prognosis of patients.

## Functional Study and Therapeutic Translation

IBD is a multifactorial disease derived from dysregulated immune responses in genetically susceptible individuals. Aberrant immunoregulation, impaired intestinal epithelial barrier, and abnormal autophagy significantly contribute to the complicated pathogenesis of IBD. Substantial evidence has demonstrated the widespread impacts of epigenetic patterns on IBD-associated signal pathways and functional changes, which facilitates a better understanding of the interactions between genetic and environmental factors, and provides an impetus for translational research on epigenetics-based therapeutics for patients with IBD. In this part, the functional impacts of epigenetic changes in the most extensively investigated pathways of IBD, and the roles of epigenetics in therapeutic translation will be discussed (**Table 3**).

**Table 3:** Functional study of epigenetic modifications in IBD.

Epigenetic modifications	Functions
**Immunoregulation**	
PAR2	Regulates the production of inflammatory cytokines, and the proliferation of intestinal myofibroblast
RUNX3	Regulates T-cell development and TNF-β signaling pathways
TRAF6, IL12B, HLA-DOB, IL16, IGHG1 and THY1	Implicate in lymphocyte development, antigen processing, and cytokine responses
miR-155	Regulates the differentiation of T helper cells and the expression of proinflammatory cytokine, inhibits the expression FOXO3a and the NF-κB signaling pathway
miR-21	Mediates Th2 cell differentiation, modulates T-cell-mediated immune responses, involves in PTEN/PI3K/Akt signaling pathways, and disrupt intestinal epithelial barrier
miR-301a, miR-20b, miR-10a, miR-18a, miR-210, miR-223, miR-155, miR-26a and miR-21	Implicate IL23/Th17 pathways
miR-146a	Modulates Treg cells, dendritic cells and NK cells, and signaling pathways related to NOD2 and TLRs
miR-192, miR-20, miR-143, miR-150, miR-122, miR-29, miR-132, miR-495, miR-512 and miR-671	Implicate NOD2 signaling pathways
miR-146a, miR-144, miR-155, miR-132 and let-7	Implicate TLR signaling pathways
miR-124, let-7, miR-125, miR-26 and miR-101	Implicates STAT3 signaling pathway
**Intestinal epithelial barrier**	
CDH1	Encodes e-cadherin and mediates adherens junctions
MDR1	Involves in transmembrane transport and functional maintenance of intestinal epithelium
miR-21	Damages tight junctions and increases the permeability
miR-200b	Prevents intestinal inflammation, and protects tight junction and paracellular permeability
miR-122a	Increases the levels of zonulin and weakens the intestinal barrier
**Autophagy**	
miR-142-3P, miR-106b and miR-93	Decrease the ATG16L1-mediated autophagic activity
miR-196	Decreases the IRGM-mediated autophagic activity

T-cell differentiation and activation, antigen processing (recognition, presentation and binding), and cytokine production are the most studied fields of immunoregulation in IBD (CD and UC). PAR2 activation displayed pro-inflammatory and anti-inflammatory effects on colon, by promoting the production of T-helper cell type 1 (Th1) cytokines (TNF-α, IL-1 and IFN-γ), and the release of calcitonin gene related peptide (CGRP) respectively (Fiorucci et al., 2001; Cenac et al., 2002). Higher methylation levels of PAR2 are associated with severe phenotypes of UC (Tahara et al., 2009b), implying that accumulated inflammation and immune dysfunction derived from PAR2 methylation might result in severe disease behaviors of UC. Besides, PAR2 is also up-regulated by TNF-α (one of the most important mediator in CD and UC), and implicated in the activation of cytosolic phospholipase A2 (cPLA2) and proliferation of intestinal myofibroblast in CD patients, thereby playing a vital role in stricture formation of CD (Christerson et al., 2009). *RUNX3* is a tumor-suppressor gene that is implicated in the pathophysiology of IBD and CRC. One of the IBD (CD and UC) susceptibility loci is located in the chromosomal region 1p36 where *RUNX3* resides (Brenner et al., 2004). *RUNX3* plays a certain role in T-cell development and TNF-β signaling pathways that are associated with the pathogenesis of both CD and UC. Studies have showed that *RUNX3* knockout mice presented over-responsiveness to antigens, over stimulation of T-cells, and spontaneous IBD (Brenner et al., 2004; Garrity-Park et al., 2010). Thus, it seems likely that RUNX3 methylation may contribute to the excessive inflammatory responses in both CD and UC. Moreover, UC–CRC cases presented much higher methylation levels of *RUNX3* than UC controls, indicating that *RUNX3* agonists might play an anti-inflammatory and anti-cancer role in clinical settings (Garrity-Park et al., 2010). Methylation modifications in other genes (*TRAF6*, *IL12B*, *HLA-DOB*, *IL16*, *IGHG1* and *THY1*) were also claimed to be either involved in T-cell or B-cell development, or implicated in antigen processing and cytokine responses, which provided a basis for drug discovery in the future (Gould et al., 2016). In addition to DNA methylation, miRNAs are also implicated in several immunoregulation processes related to IBD. Overexpression of miR-155 mediates a bias towards Th1 differentiation, while loss of miR-155 is prone to Th2 differentiation (Kalla et al., 2015). Previous evidence has suggested that CD was associated with Th1 and Th17 cytokine patterns, whereas UC was thought to be correlated with Th2-mediated inflammation (Brand, 2009). Up-regulated miR-155 exerts a pro-inflammatory effect by inhibiting the expression of Forkhead box O3 (FOXO3a) and therefore promotes the expression of inflammatory cytokines, and IBD-associated NF-κB signaling pathway. However, a deficiency in miR-155 shows a protective effect on experimental colitis by diminishing the expression of proinflammatory cytokine (TNF-α, IL-6, IL-12, IL-17, and IFN-γ), weakening the activation of T-cells, and repressing the Th1-mediated immune responses (Wan et al., 2016). miR-21 is overexpressed in patients with IBD. It mainly mediates UC-associated pathophysiological processes, including Th2 cell differentiation, T-cell-mediated immune responses, PTEN/PI3K/Akt signaling pathway, and the disruption of intestinal epithelial barrier (Kalla et al., 2015; Moein et al., 2019). miR-21 knockout mice with experimental dextran sulfate sodium (DSS) colitis showed an improved survival rate and less inflammation and injury in tissues when compared with wild type mice (Shi et al., 2013). Taking this into consideration, miR-21 inhibition may be a promising therapeutic target for UC patients. In addition, miR-21 also plays a central role in IL23/Th17 axis. IL23/Th17 signaling pathway has been reported to contribute greatly to the pathogenesis of CD. GWAS have identified several susceptibility genes of CD (*IL23R*, *IL12B*, *JAK2*, *STAT3*, *CCR6* and *TNFSF15*) that were involved in IL23/Th17 signaling pathway. Th17 is a novel kind of proinflammatory cell, and is implicated in the intestinal inflammation of CD by promoting the production of proinflammatory cytokines (IL17A, IL17F, IL21, IL22 and IL26) and chemokines (CCL20) (Brand, 2009). Other miRNAs implicated in IL23/Th17 pathways include miR-301a, miR‐20b, miR‐10a, miR‐18a, miR‐210, miR‐223, miR‐155, miR‐26a and miR‐21 (He et al., 2016; Moein et al., 2019). Recent studies have identified a direct and positive regulatory effect of miR-301a on the differentiation of Th17 cells and the production of proinflammatory cytokines through down regulation of Smad Nuclear Interacting Protein 1 (SNIP1) (He et al., 2016). In this respect, blockers of miR-301a may be a promising therapeutic intervention for CD patients. miR-146a involves the modulation of Treg cells, dendritic cells and NK cells, and signaling pathways related to NOD2 and Toll-like receptors (TLRs) (Kalla et al., 2015; Moein et al., 2019). NOD2 and TLRs are most integral parts in the pathogenesis of IBD, especially for CD. NOD2 can recognize the bacteria-derived muramyl dipeptide (MDP), and activate the NF-κB and caspase3 signaling pathways, and then, produces proinflammatory cytokines and regulates the innate and adaptive immunity of intestine (Kullberg et al., 2008). Moreover, it is also involved in the maintenance of the mucosal antibacterial barrier by regulating the expression of alpha-defensin and beta-defensin (Wehkamp et al., 2004; Voss et al., 2006). Thus, NOD2 variant/deficiency is a certain contributor to the development of CD. Existing data revealed that miR-192 and miR-20 showed inhibitory effects on the expression of *NOD2*, while miR‐143 and miR‐150 influenced the *NOD2* by targeting the important mediators of *NOD2* signaling pathway. miR-122, miR-29, miR‐132, miR‐495, miR‐512 and miR‐671 are other miRNAs associated with the *NOD2* signal and IBD pathogenesis. It’s noteworthy that miR-122 designed for Hepatitis C infections is the first miRNA-based therapies in human clinical trials, which hold a great promise for future clinical research in other diseases such as IBD (Janssen et al., 2013). Additionally, an agent targeting miR-29 was also undergoing phase II clinical trials, with the aim of preventing tissue fibrosis. With regard to TLRs, TLR4 is largely activated by the lipopolysaccharide (LPS)-LPS-binding protein (LBP)-CD14 complex, and then triggers the NF-κB signaling pathway and promotes the production of proinflammatory cytokines (Chow et al., 1999). Besides, it is also proposed that TLR4-mediated signals can be modulated by NOD2, and NOD2 mutations can damage the cross-tolerance between NOD2 and TLR4, thus increasing the risk of CD (Kullberg et al., 2008). Available evidence indicated that miR-146a targets TLR4 signaling pathways and plays an anti-inflammatory role in CD, while miR‐144 targets TLR2 and serves as a pro-inflammatory marker (Kalla et al., 2015; Moein et al., 2019). Other miRNAs associated with TLR signaling pathways include miR-155, miR‐132 and let-7 (Koukos et al., 2013; Moein et al., 2019). Signal transducer and activator of transcription 3 (STAT3) signaling pathway is another vital transduction pathway, which is responsible for prolonging the survival of pathogenic T cells, and exacerbating inflammatory responses, therefore contributing to the pathogenesis of both CD and UC (Sugimoto, 2008). Koukos et al. (2013) have indicated that miR-124, let-7, miR-125, miR-26, and miR-101 could decrease STAT3 phosphorylation, and thereby suppress the inflammatory responses in UC patients. Amongst these miRNAs, miR-124 outperformed others, and showed a decreased level in active states in comparison to quiescent states of UC patients. Collectively, epigenetic patterns show a widespread influence on immunological functions associated with IBD, which provides some new druggable receptors for novel therapeutics. Some miRNA agonists and antagonists have been developed and successfully applied in mouse models of colitis. For example, treatment with miR-155 antagonists alleviates the inflammatory responses in DSS-induced colitis mouse model (Lu et al., 2017). He et al. (2016) devised miR-301a antisense oligonucleotide and administrated it in trinitrobenzene sulphonic acid (TNBS)-induced mouse colitis model. As a result, a notable decrease in IL-17A cells and pro-inflammatory cytokines has been noticed in the inflamed tissues. Remarkable results gained in animal studies provide a strong driving force for translational studies and for developing novel epigenetics-based therapeutics for patients with IBD.

The impairment of intestinal epithelial barrier is one of the most critical pathogenic factors for IBD, especially for UC. Accumulated evidence has revealed that intestinal epithelial barrier has an established effect on defending against pathogenic microorganism invasion and colonization, preventing toxin translocation, and maintaining immune balance (Latiano et al., 2008; Consortium et al., 2009). IBD patients and even individuals at high risk of developing IBD could present impaired cell-cell junction and increased intestinal permeability (Wolters et al., 2011). Several genes including *CDH1*, *LAMB1*, *HNF4A* and *MYO9B* that are involved in the maintenance of epithelial barrier function have been claimed to be risk genes of UC (Latiano et al., 2008; Wolters et al., 2011). *CDH1* gene is located within the IBD1 locus, and encodes e-cadherin and mediates adherens junctions of colonic epithelia. Its decreased expression level and increased methylation status have been found in active UC and CRC tissues, suggesting a possibility of using CDH1 methylation marker to classify active disease from inactive disease, and CRC from healthy controls (Saito et al., 2011; Cooke et al., 2012). Similar to *CDH1*, *MDR1* gene also encompasses susceptibility loci of UC. It is involved in transmembrane transport and functional maintenance of intestinal epithelium (Tahara et al., 2009a). Mice lacking *MDR1a* gene spontaneously suffered from UC-like intestinal inflammation (Panwala et al., 1998; Ho et al., 2005). And the expression levels of *MDR1* in DSS-induced colitis mouse model and UC patients were reduced in comparison to healthy controls (Ho et al., 2005). Higher methylation levels of *MDR1* in inflammatory tissues relative to normal tissues of UC patients further supported the protective effects of *MDR1* in intestinal epithelium (Tahara et al., 2009a). In addition to methylation profiles, different kinds of miRNAs also showed their protective or destructive function in intestinal barrier. miR-21 damages tight junctions and increases the permeability of intestine through targeting RhoB and PTEN/PI3K/Akt pathways (Yang et al., 2013; Moein et al., 2019). It also regulates the malignant phenotypes of CRC by reducing the phosphatase and tensin homolog (PTEN), indicating a possibility of evaluating the CRC transformation and progression by it. Whereas, miR-200b exerts a protective effect on intestinal inflammation, tight junction, and paracellular permeability by down regulating the expression of IL-8 secondary to the activation of TNF-α, and inhibiting the destabilization of claudin 1 and zonula occludens-1 (ZO-1) (Shen et al., 2017). miR-122a weakens the intestinal barrier by targeting the EGFR pathways and increases the levels of zonulin, thereby increasing intestinal permeability, promoting pathogen invasion, and aggravating intestinal inflammation. Additionally, miR‐191a, miR‐93, miR‐150, miR‐675 and miR‐874 also can affect functions of intestinal epithelial barrier (Moein et al., 2019). Altogether, diverse epigenetic modifications exert facilitating or damaging effects on intestinal epithelial barrier, which proves a novel avenue for IBD treatment. Producing antagomirs or miRNA mimics that are involved in regulation of intestinal epithelial barrier may be fruitful in future. Unfortunately, there is still no ongoing trial targeting these miRNAs for IBD. Instead, a trail targeting miR-122, miR-196 and miR-34 for glioblastoma multiforme and metastatic breast cancer is in the preclinical phase. Thus, continuous efforts are required to achieve translational research.

Successfully unveiling the contribution of autophagy to the pathogenesis of IBD has been a milestone achievement in the field of IBD research. Autophagy is dynamic cellular recycling process that is responsible for the degradation of abnormal cytoplasmic component (Kim and Lee, 2014). Recent studies have claimed that autophagy greatly affected the pathogenesis of IBD (especially of CD) by modulating the process of pathogen clearance, antimicrobial peptide secretion, inflammatory response, antigen presentation, and the endoplasmic reticulum (ER) stress response (Hooper et al., 2017; Iida et al., 2017). *ATG16L1*, *NOD2* and *IRGM* are the most investigated autophagy-related genes in CD. The interplay between autophagy-related genes and different miRNA offers deep insights into pathophysiological mechanisms of CD. miR‐142‐3P, miR‐106b and miR‐93 are claimed to target *ATG16L1*, while miR-196 is involved in IRGM-mediated autophagy. miR-142-3p directly reduces the mRNA and protein levels of ATG16L1, thereby decreasing starvation-induced and L18-MDP-induced autophagic activity (Zhai et al., 2014). A hallmark study revealed that miR-106b was increased while ATG16L1 was decreased in intestinal tissues of active CD patients in comparison to controls. miR-106b and miR-93 were claimed to target *ATG16L1* mRNA, thereby inhibiting the expression levels of ATG16L1 and damaging autophagy-mediated bacteria eradication. Antagonists for miR-106b and miR-93 facilitated the formation of autophagosomes, thus, alleviating intestinal inflammation (Lu et al., 2014). As for miR-196, several studies have seen an increase of it in patients with CD (Zhang et al., 2018). Overexpressed miR-196 can down regulate the protective variant (c.313C) in *IRGM*, thereby causing a disturbance in the regulation of *IRGM*. As a result, the expression levels of *IGRM* and efficacy of autophagy are diminished, and the growth of CD-associated intracellular bacteria (Adherent Invasive *Escherichia coli*, AIEC) is out of control, leading to an increased risk of developing CD (Brest et al., 2011). On the basis of this, miRNA-based regulation in IRGM-dependent autophagy may play a certain role in CD. On the other hand, it may open up a new research direction in autophagy and drug development of CD. Many approved drugs including corticosteroids, aminosalicylates, thiopurines, cyclosporin, tacrolimus and anti-TNF biologics exert their therapeutic effects by modulating signaling pathways that are often directly or indirectly associated with autophagy, but drugs targeting miRNA are still lacking (Hooper et al., 2017). Developing miRNA-based pharmacotherapy that specifically targets autophagy represents a promising therapeutic option for CD patients. However, the cell-type-specific feature of autophagy makes it difficult to do autophagy-targeted drug discovery (Hooper et al., 2017). Further research is needed to resolve this difficulty.

Although histone alterations have been less studied in IBD, some studies still suggest its potential roles in disease. Acetylation of H4 was upregulated in inflamed tissues and Peyer’s patches of CD patients and DSS-induced colitis models, highlighting its pro-inflammatory effects in colon (Tsaprouni et al., 2011). Treated with histone deacetylase (HDAC) inhibitors, mice consequently showed an apparent attenuation in intestinal inflammation. It’s important to note that HDAC inhibitors have multiple targets including some other non-histone targets (TLR4, β-defensin 2, STAT3, P53 etc.), and are involved in a variety of IBD-associated signaling pathways such as NF-κB and Foxp3 transduction pathways (Tsaprouni et al., 2011; Ventham et al., 2013). In addition, tight links between lncRNA signatures and IBD-related inflammatory responses have also been described in several studies (Padua et al., 2016). Indeed, histone alterations and lncRNAs are important contributors for IBD activity, but associations with disease susceptibility, behaviors and prognosis are yet to be elucidated in the near future. Although some drugs targeting HDAC are used in clinical trials, most are designed for hematological malignancies and solid tumors. Therefore, annotation of the therapeutic utility of histone alterations and lncRNAs in IBD is also in dire need.

Dramatic success in development and application of biologics to IBD has brought IBD therapy into a new horizon. However, primary non-responders and secondary non-responders to biologics have still remained. Adverse reactions and high economic burden of existing biologic agents are real challenges in IBD treatment, highlighting the need of exploring new therapeutic strategies with good efficacy and less side effects for IBD patients. In-depth understanding of roles of epigenetic alterations in IBD susceptibility, activity, behaviors, and CRC provides a powerful driving force for the development of epigenetics-based therapeutics. Whereas, the process of therapeutic translation is in slow progress. Drug development as a whole is also being faced with numerous challenges. Firstly, currently used DNA methylating/demethylating agents show poor efficiency as a therapeutic modality due to the poor chemical stability, low specificity, and strong secondary effects (Gros et al. 2012). Azacitidine and decitabine are the two drugs approved by the US Food and Drug Administration (FDA) for myelodysplastic syndrome and acute myeloid leukemia, with common side effects such as hepatotoxicity and nephrotoxicity (Issa and Kantarjian, 2009). Constructing highly efficient and selective DNA methylation-based therapeutics is required. Secondly, since gut microbiota can regulate histone acetylation and methylation patterns of intestine, and epigenetic changes are cell/tissue-specific and time-dependent, identifying the biological impacts of gut microbiota on epigenetic patterns, and the etiological contributions of epigenetic modifications to gastrointestinal disorders remain difficult (Aleksandrova et al., 2017). Thirdly, delivery technologies for miRNA modulators to specific cell types and tissues, and off-target effects of miRNA-based therapeutics pose a major challenge for researchers. Fourthly, definite miRNA targets, exact mechanisms of action, and functional impacts of miRNAs should also be taken into account. In addition, more efforts are needed to annotate the long-term effects and pharmacokinetics, pharmacodynamics and pharmacogenetics of miRNA mimics or antagomirs *in vivo* (van Rooij and Kauppinen, 2014). Overcoming these difficulties at the earliest is of paramount importance.

## Conclusions

IBD is an extremely complicated disease and poses a big challenge for physicians with regard to diagnosis and management of patients. In the era of precision medicine, we advocate that diagnosis, treatment and surveillance of diseases must be based on individual genetic markers, phenotypic characteristics, and psychosocial features (Chow et al., 2018). Substantial progress has been made in the genetic study of IBD, with numerous IBD-associated susceptibility loci identified. However, the identified genetic factors can explain only a small portion of overall disease variance, highlighting the need of uncovering the role of other factors such as epigenetic modifications in the occurrence and development of IBD. Epigenetic changes can mediate the interaction between genetics and environment, providing some critical information related to IBD pathogenesis. Recent years have seen a substantial advancement in epigenetics of IBD, particularly with relation to DNA methylation and miRNAs. Significant associations between epigenetic modifications and disease susceptibility, activity, behavior, and IBD-associated CRC have been shown in numerous studies, providing in-depth insights into the molecular basis of IBD, and additional diagnostic and monitoring tools for IBD patients. Several DNA methylation/miRNA-based panels for diagnosis and differential diagnosis, disease activity assessment, disease behavior evaluation, and CRC detection and surveillance have been developed, with good sensitivity, specificity and accuracy. Epigenetic markers are also candidate indicators for the selection of therapeutic methods and the prediction of therapeutic response. Functional studies have showed the significant impacts of epigenetic changes on the IBD-related immunoregulation, maintenance of intestinal epithelial barrier, and modulation of autophagy, notably in the most extensively investigated filed such as T-cell differentiation, IL23/Th17 and STAT3 signaling pathways, and intestinal permeability, which further enhance our knowledge of the biological processes of IBD. Based on the crucial contributions to IBD, pharmacological modulation of epigenetic patterns provides possibilities of therapeutic translation for the future clinical applications. However, current clinical trials or preclinical trials are focused on cancer treatment and obtain some preliminary achievements, providing a glimpse of translational potential of IBD-associated epigenetic modifications. Epigenetic research of IBD is in its infancy, and there are still some challenges to address. More endeavors are needed to compare the performance of epigenetic surrogates with classical and emerging markers, and to establish more robust diagnostic and monitoring panels comprising of different-class of markers. Continuous efforts should also be made to construct highly efficient and selective therapeutics, identify targets and functional impacts of epigenetic modifications, improve delivery technologies for miRNAs, and elucidate biological effects of gut microbiota on epigenetic patterns. Moreover, considering that histone modifications and nucleosome positioning and other non-coding RNAs such as siRNA, piRNA and lncRNA are less studied in the field of IBD, further efforts should be made to identify the roles of these epigenetic changes in the pathogenesis of IBD. Therefore, it can be concluded that epigenetics plays a critical role in the pathogenesis of IBD, and holds a promise for disease diagnosis and surveillance, as well as for risk prediction and therapeutic innovation.

## Author Contributions

ZZ and HZ outlined the overall manuscript, and ZZ drafted the manuscript; HZ supervised the preparation of the draft and edited it. AM helped write, proofread, and edit the final manuscript.

## Funding

This Work was Supported by the National Natural Science Foundation of China [No. 81570502] and by the 1.3.5 Project for Disciplines of Excellence, West China Hospital, Sichuan University [Grant Number: ZYJC18037].

## Conflict of Interest

The authors declare that the research was conducted in the absence of any commercial or financial relationships that could be construed as a potential conflict of interest.
